# LY2874455 and Abemaciclib Reverse FGF3/4/19/CCND1 Amplification Mediated Gefitinib Resistance in NSCLC

**DOI:** 10.3389/fphar.2022.918317

**Published:** 2022-06-23

**Authors:** Dongcheng Liu, Hongguang Liu, Jiadi Gan, Shinuan Zeng, Fuhua Zhong, Bin Zhang, Zhe Zhang, Siyu Zhang, Lu Jiang, Guangsuo Wang, Yixin Chen, Feng-Ming Spring Kong, Wenfeng Fang, Lingwei Wang

**Affiliations:** ^1^ Department of Respiratory and Critical Care Medicine, Shenzhen Institute of Respiratory Diseases, The Second Clinical Medical College of Jinan University, The First Affiliated Hospital of Southern University of Science and Technology, Shenzhen People’s Hospital, Shenzhen, China; ^2^ Shenzhen Aier Eye Hospital Affiliated to Jinan University, Shenzhen, China; ^3^ Department of Clinical Medical Research Center, The Second Clinical Medical College of Jinan University, The First Affiliated Hospital of Southern University of Science and Technology, Shenzhen People’s Hospital, Shenzhen, China; ^4^ Integrated Chinese and Western Medicine Postdoctoral Research Station, Jinan University, Guangzhou, China; ^5^ Department of Laboratory Medicine, Huazhong University of Science and Technology Union Shenzhen Hospital (Nanshan Hospital), Shenzhen, China; ^6^ Department of Medical Oncology, State Key Laboratory of Oncology in South China, Collaborative Innovation Center for Cancer Medicine, Sun Yat-sen University Cancer Center, Guangzhou, China; ^7^ Department of Thoracic Surgery, The First Affiliated Hospital of Southern University of Sciences and Technology, Shenzhen People’s Hospital, Shenzhen, China; ^8^ Department of Oncology, The Second Clinical Medical College of Jinan University, The First Affiliated Hospital of Southern University of Science and Technology, Shenzhen People’s Hospital, Shenzhen, China; ^9^ Department of Clinical Oncology, The University of Hong Kong-Shenzhen Hospital, Shenzhen, China

**Keywords:** gefitinib resistance, FGF3/4/19/CCND1 amplification, NSCLC, LY2874455, abemaciclib

## Abstract

Non-small cell lung carcinoma (NSCLC) patients who initially received tyrosine kinase inhibitor (TKI) therapy often acquired resistance *via* multiple complex mechanisms. The amplification of FGF3/4/19/CCND1 on chromosome 11q13 was found in many cancers with TKI resistance. However, the role of these amplifications in TKI-resistant NSCLC remains uncovered. Here, we generated the FGF3/4/19/CCND1 amplification model in the NSCLC cell lines PC-9 and HCC827. Upregulation of FGF3/4/19/CCND1 strongly promoted cell proliferation and gefitinib resistance in NSCLC cells. To find out the potential therapeutic strategies, we screened the combination of inhibitors against the FGF/FGFR signaling pathway and the CCND1/CDK4 complex and revealed that gefitinib combined with LY2874455 and abemaciclib exhibited the most effective inhibition of resistance *in vitro* and *in vivo*. Mechanistically, FGFs/CCND1 activated the MAPK pathway, which was abolished by the combination drugs. Our study provides a rationale for clinical testing of dual targeting FGFR and CCND1 with LY2874455 and abemaciclib in NSCLC patients who harbored FGF3/4/19/CCND1 amplification.

## Introduction

Lung cancer is one of the leading causes of cancer-related deaths worldwide (Siegel et al., 2021). Non-small cell lung carcinoma (NSCLC), the main type of lung cancer, accounts for 83% of all lung cancer cases (Siegel et al., 2021). The 5-year survival rate of NSCLC patients is only approximately 26% (Goldstraw et al., 2016). Previously chemotherapy has been the primary treatment option. As new therapeutics and technologies are developed in the era of precision medicine, targeted therapy is used to achieve a better prognosis for patients with advanced-stage NSCLC (Hirsch et al., 2017; Sharifi et al., 2022).

Epidermal growth factor receptor (EGFR) is a transmembrane glycoprotein that belongs to the ErbB family of RTKs, which includes ErbB-1 (EGFR), ErbB-2 (HER2/neu), ErbB-3 (HER3), and ErbB-4 (HER4) (Kyriakopoulou et al., 2018). The abnormal activation of EGFR can stimulate the downstream intracellular signaling pathways such as phosphoinositide 3-kinase (PI3K)-AKT, mammalian target of rapamycin (mTOR), and mitogen-activated protein kinase (MAPK), which are the critical regulators of cell proliferation, differentiation, migration, and apoptosis (Liu et al., 2018). In NSCLC patients, EGFR signaling is frequently upregulated due to the amplification or mutation of the EGFR gene (Levantini et al., 2022; Tan and Tan, 2022). Due to the geographical diversity, the prevalence of EGFR mutations in Asian NSCLC patients is much higher (40–60%) than in Caucasians (10–15%) (Tan et al., 2016). The two “classical” EGFR mutations, small in-frame deletions in exon 19 and single amino acid substitution (L858R) in exon 21, collectively account for about 90% of known activating EGFR mutations (Tan and Tan, 2022). EGFR tyrosine kinase inhibitors (TKIs) such as gefitinib are the commonly applied targeted therapy for patients with these EGFR mutations (Westover et al., 2018; Murtuza et al., 2019). Recent studies have shown that gefitinib significantly increased the overall survival of NSCLC patients (Zhao et al., 2017; Ramalingam et al., 2020). However, most patients will inevitably acquire TKI resistance as treatment proceeds, which largely limits the overall survival of patients with NSCLC (Westover et al., 2018).

Two central mechanisms are involved in the resistant process: 1) the genetically novel EGFR mutations that allow the constant activation of EGFR signaling even with TKI therapy (Wu and Shih, 2018); 2) activation of bypass survival tracks through other RTKs or alternative downstream (Liu et al., 2018). The effective treatments of on-target resistant mutations such as T790M or C797S mutation in EGFR have been developed (Terp et al., 2021). Unfortunately, a substantial percentage of resistant cases remain mechanistically unexplained, which requires further investigation.

The FGF/FGFR system is comprised of four receptors (FGFR1-4) and 19 ligands (FGFs) (Xie et al., 2020). FGFRs are the tyrosine kinase receptors consisting of an intracellular tyrosine-kinase domain and an extracellular ligand-binding domain (Katoh, 2019). FGF/FGFR signaling network plays a critical role in cell proliferation, survival, migration, and drug resistance in various cancers (Hanker et al., 2017; Ghedini et al., 2018; Xie et al., 2020). The binding of FGFs to FGFRs leads to receptor dimerization which enables the cytoplasmic kinase domains to transphosphorylate one another at specific tyrosine residues (Xie et al., 2020). Eventually, the downstream signalings are triggered through the RAS-MAPK-PI3K-AKT or mTOR pathway that regulates cell roliferation, differentiation, and survival (Xie et al., 2020). Increasing studies have identified novel drugs for targeting FGF/FGFR pathway (Liu et al., 2020; Makvandi et al., 2021). Nevertheless, the role of the FGF/FGFR network in TKI-resistant NSCLC is poorly understood, resulting in the lack of effective clinical treatment in cancers carrying dysregulation of FGF/FGFR signaling.

In this study, we observed that FGF3/4/19/CCND1 amplification strongly promoted gefitinib resistance *in vitro and in vivo*. The combination of LY2874455 and abemaciclib was necessary to completely inhibit the growth of tumor harbored FGF3/4/19/CCND1 amplification.

## Materials and Methods

### Patients and Clinical Information

Tumor samples were obtained from Sun Yat-sen University Cancer Center from 2017 to 2021. The clinical information of patients with FGF3/4/19/CCND1 amplification among 285 TKI-resistance patients was listed in Table 1. These patients were collected after resistance to treatment of EGFR TKIs. These patients had various degrees of extrathoracic metastasis. The present study was approved by the Ethics Committee of Sun Yat-sen University Cancer Center. All study procedures were performed according to the Declaration of Helsinki ethical principles. Informed consent was obtained from the patients.

**TABLE 1:** Clinicopathological characteristics of patients with FGF3/4/19/CCND1 amplification among 285 TKI-resistance patients

No. of patients (%)			
	First- or second-generation EGFR-TKIs resistance	Third-generation EGFR-TKIs resistance	Total
Age (years)			
≥60	5 (62.5%)	2 (40.0%)	7 (53.8%)
<60	3 (37.5%)	3 (60.0%)	6 (46.2%)
Gender			
Male	3 (37.5%)	1 (20.0%)	4 (30.8%)
Female	5 (62.5%)	4 (80.0%)	9 (69.2%)
Smoking			
Smoker	0 (0.0%)	0 (0.0%)	0 (0.0%)
Non-smoker	8 (100.0%)	5 (100.0%)	13 (100.0%)
Sample type			
Tumor tissue	3 (37.5%)	1 (20.0%)	4 (30.8%)
ctDNA	1 (12.5%)	3 (60.0%)	4 (30.8%)
Others*	4 (50.0%)	1 (20.0%)	5 (38.5%)
TNM stage			
M1a/b	4 (50.0%)	1 (20.0%)	5 (38.5%)
M1c	4 (50.0%)	4 (80.0%)	8 (61.5%)
Total	**8**	**5**	**13**

*Pleural effusion or cerebrospinal fluid or NA.

### Cell Culture and Compounds

PC-9, HCC827, and 293T cells were purchased from the American Type Culture Collection (ATCC). These cells were authenticated using short tandem repeat (STR) (Igebio, Guangzhou, China). PC-9 and HCC827 cells were cultured in RPMI-1640 (Hyclone, Thermo Scientific) medium supplemented with 10% fetal bovine serum (Hyclone, Thermo Scientific) at 37°C in an incubator with 5% CO_2_. 293T cells were cultured in Dulbecco’s modification of Eagle’s medium (Hyclone, Thermo Scientific) supplemented with 10% fetal bovine serum (Hyclone, Thermo Scientific) at 37°C in an incubator with 5% CO_2_. All cell lines were confirmed with negative mycoplasma contamination. All compounds were purchased from Selleck Chemicals. Compounds were prepared as 10.0 mM stock solutions in DMSO and stored at −20°C. Independent aliquots of stock solutions were thawed prior to use in each experiment.

### Plasmid Construction

To construct the plasmid pLenti-CMV-FGF3-FGF4-FGF19-hygromycin, the sequence of FGF3-p2A-FGF4-t2A-FGF19 was synthesized and inserted into pLenti-CMV-MSC-hygromycin backbone. To construct the pLV-CCND1-puromycin plasmid, CCND1 cDNA was amplified by PCR from 293T cells and inserted into pLV-EF1α-MSC-puromycin backbone *via* the EcoRI/XbaI sites.

### Lentivirus Production and Transduction

Lentivirus constructs with packing plasmids psPAX2 and pMD2.G were co-transfected into 293T cells. Viruses were collected at 48 h and 72 h after transfection and then added to PC-9 or HCC827 cells with polybrene (8 μg/ml, Sigma). Forty-eight hours after infection, puromycin (2 μg/ml) or/and hygromycin (200 μg/ml) were added to the culture medium for stable cell selection. Real-time quantitative PCR(RT-qPCR) and Western blot were performed to determine the expression level.

### RNA Extraction and Real-Time Quantitative PCR Assays

Total RNA was extracted from cells using TRIZOL Reagent (Invitrogen, United States), and cDNA was synthesized from 1 μg of RNA with the One-Step gDNA Removal and cDNA Synthesis SuperMix (Transgen, Beijing, China) as recommended by the manufacturer. Real-time quantitative PCR reactions for the quantification of gene expression were performed with the Bio-Rad iQ5 Real-time PCR system. The primer sequences used in this study are listed in Table 2.

**TABLE 2:** Primers used for real-time quantitative PCR.

Gene	Sequences (5′ → 3′) (forward)	Sequences (5′ → 3′) (reverse)	GenBank accession number
FGF3	GGC​GTC​TAC​GAG​CAC​CTT​G	CAC​CTC​CAC​TGC​CGT​TAT​CTC	NM_005247.4
FGF4	GGGCGTGGTGAGCATCTT	TTG​TAG​GAC​TCG​TAG​GCG​TTG	NM_002007.4
FGF19	TCT​CCC​TGA​GCA​GTG​CCA​AAC	TCC​GGT​GAC​AAG​CCC​AAA​T	NM_005117.3
CCND1	GGTGGCAAGAGTGTGGAG	CCT​GGA​AGT​CAA​CGG​TAG​C	NM_053056
GAPDH	TGA​CTT​CAA​CAG​CGA​CAC​CCA	CAC​CCT​GTT​GCT​GTA​GCC​AAA	NM_001256799.3

### Western Blot

Total protein was extracted and protein concentration was determined with the BCA Protein Assay Kit (Pierce, Rockford, IL, United States). Equivalent amounts of protein samples were uploaded and separated by SDS-PAGE and then electrotransferred to polyvinylidene difluoride (PVDF) membranes (Millipore Corp, Atlanta, GA, US). The membranes were blocked in 5% non-fat dry milk powder at room temperature for 1 h, and then incubated overnight at 4°C with primary antibodies: anti-FGF3 (1:1000 dilution, Abclonal, Cat # A19052), anti-FGF4 (2 µg/ml, Abclonal, Cat # ab65974), anti-FGF19 (1:1000 dilution, Abcam, Cat # ab85042), anti-EGFR (1:1000 dilution, Abclonal, Cat # A11351), anti-phospho-EGFR-Y1068 pAb (1:1000 dilution, Abclonal, Cat # AP0301), anti-mTOR (1:1000 dilution, Abclonal, Cat # A11354), anti-phospho-mTOR (2448) (1:1000, Abcam, ab109268), anti-p44/42 MAPK (Erk1/2) (1:1000, CST, 4695S), anti-phospho-p44/42 MAPK (Erk1/2) (Thr202/Tyr204) (1:2000, CST, 4370S), anti-GAPDH (1:1000 dilution, CST, Cat#5174S), anti-beta-tubulin (1:1000 dilution, CST, Cat#2146). Membranes were then incubated with HRP-conjugated secondary antibodies at room temperature for 1 h. The signals of bands were detected by ECL reagents.

### Cell Viability Assay

Cells were seeded in 96-well assay microplates at a density of 2,000 cells per well in a total volume of 100 µl per well and incubated at 37°C, 5% CO_2_ overnight. To determine the cell proliferation, the CCK8 assay was carried out at the indicated time. For IC 50 determination, cells were seeded into 96-well plates and incubated in 10-fold escalating concentrations of gefitinib and indicated compounds for 72 h. Proliferation was assessed by the CCK8 assay (MedChemExpress, NJ, United States). IC50 values were calculated using Graphpad Prism software using non-linear regression curve fit analysis and are reported as the mean ± SD of three independent experiments.

### Colony Formation Assay

Cells were seeded into 24-well plates at a density of 300 cells per well and incubated at 37°C, 5% CO_2_ overnight and then the medium was replaced with fresh media containing 10% FBS and exposed to gefitinib and indicated drugs, and the medium was replaced every 3 days. The colonies were fixed with methanol and stained with 0.1% crystal violet for 15 min. Each experiment was performed in triplicate.

### *In Vivo* Tumorigenesis Assay

Experimental procedures were performed in accordance with the Guide for the Care and Use of Laboratory Animals (National Institutes of Health Publication No. 80-23) and according to the institutional ethical guidelines for animal experiments. Female BALB/c nu/nu mice (4–5 weeks old) purchased from GemPharmatech Co., Ltd. (Nanjing, China) were housed under specific pathogen-free conditions. Mice were randomly divided into four groups with six mice in each group. PC-9-4X cells (5 × 10^6^ cells/mice) were injected subcutaneously into the flanks of mice. Tumors were allowed to grow for 10 to 20 days until they reached a minimum volume of 200 mm. Mice were treated by oral gavage with indicated inhibitors (100 mg/kg Gefitinib or/and 3 mg/kg LY2874455 or/and 50 mg/kg abemaciclib) twice a week for 12 days. The length (L) and width (W) of tumor xenografts were measured at a 2-day interval with a Vernier caliper. Tumor volumes were calculated (V = W2 × L/2). The tumors were excised and imaged and weighted at the end of the experiment.

### Statistical Analysis

All data were expressed as mean ± standard deviation (SD). Statistical analysis was performed using the GraphPad PrismVer. 8.01 (GraphPad Software Inc., La Jolla, CA). Student’s t-tests were used to evaluate the differences between two comparison groups, and one-way or two-way ANOVA was used for multiple-group comparisons. A *p*-value of <0.05 was considered statistically significant.

## Results

### Frequency of FGF3/4/19/CCND1 Amplification in NSCLC Patients With TKI-Resistant Tumor

Amplification of FGF3/4/19 and CCND1 has been revealed in various cancers (Hanker et al., 2017; Li et al., 2020). However, it has not been determined in TKI resistance NSCLC. Whole exome sequencing (WES) was performed for NSCLC samples from TKI resistance patients. Of the 223 patients with first- or second-generation TKI (including gefitinib, erlotinib, icotinib, and afatinib) resistance in the cohort, 2.2% (5/223) of patients had FGF3/4/19 amplification and 3.1% (7/223) carried CCND1 amplification (Supplementary Figure S1A). Patients with third-generation TKI (osimertinib) resistance had a higher rate of FGF3/4/19 [6.5% (4/62)] and CCND1 [8.1% (5/62)] amplification in the cohort (Supplementary Figure S1B). After combining the two cohorts, we observed that 3.2% (9/285) of patients had FGF3/4/19 amplification and 4.2% (12/285) had CCND1 amplification (Supplementary Figure S1C).

### FGF3/4/19/CCND1 Promotes Gefitinib Resistance

**FIGURE 1:**
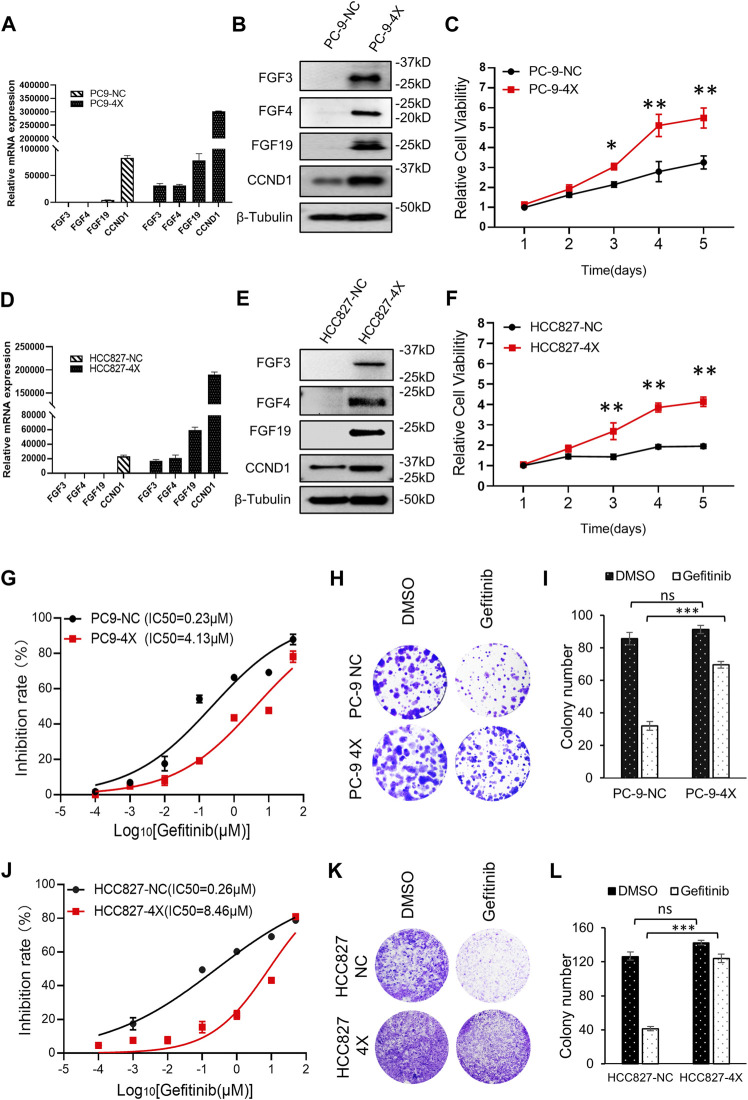
FGF3/4/19/CCND1 promotes gefitinib resistance. **(A,B)** PC-9 cells were transduced with FGF3/4/19/CCND1(4X) or empty vector (negative control, NC) by lentivirus transduction. The gene expressions were confirmed by qRT-PCR **(A)** and Western blot **(B)**. Error bars indicated standard deviation (SD), *n* = 3. **(C)** Cell viability assay. PC-9 cells were seeded in 96-well plates (2,000 cells/well). CCK8 assay was applied to measure the cell viability (*p* values reflected comparison to the control samples of indicated time points.*p < 0.05. **p < 0.01). **(D,E)** HCC827 cells were transduced with FGF3/4/19/CCND1(4X) or empty vector (negative control, NC) by lentivirus transduction. The gene expressions were confirmed by qRT-PCR **(D)** and Western blot **(E)**. Error bars indicated SD, *n* = 3. **(F)** Cell viability assay. HCC827 cells were seeded in 96-well plates (2,000 cells/well). CCK8 assay applied in indicated time to measure the cell viability (*p* values reflect comparison to the control samples of indicated time points.*p < 0.05. **p < 0.01). **(G)** The inhibition rate of gefitinib in PC-9 cells was measured by CCK8 assay following gefitinib treatment for 72 h. Each experiment was performed in triplicate. **(H)** Colony formation assay. Cells were treated with DMSO or gefitinib for 14 days. The remaining cells were stained with 0.1% crystal violet and photographed. Each experiment was performed in triplicate. Representative wells were shown. **(I)** Colonies of remaining cells were counted. Error bars indicated SD. **(J)** Inhibition rate of gefitinib in HCC827 cells was measured by CCK8 following gefitinib treatment for 72 h. Each experiment was performed in triplicate. **(K)** Colony formation assay. Cells were treated with DMSO or gefitinib for 14 days. The remaining cells were stained with 0.1% crystal violet and photographed. Each experiment was performed in triplicate. Representative wells were shown. **(L)** Colonies of remaining cells were counted. Error bars indicated SD (***p < 0.001, student’s t-test).

Previously studies have shown that FGF3/4/19/CCND1 amplification caused enhanced expression of these genes (Hanker et al., 2017; Li et al., 2020). NSCLC cell lines PC-9 and HCC827, which were sensitive to gefitinib, expressed undetectable amounts of FGF3, FGF4, FGF19, and low levels of CCND1 (Figure 1A). To mimic the amplification of FGF3/4/19/CCND1, we constructed co-expression lentivirus plasmids (see Materials and Methods) and generated FGF3/4/19/CCND1 overexpression stable cell lines from PC-9 and HCC827 cells (designated as PC-9-4X and HCC827-4X). After confirming the enhanced expression of these genes (Figures 1A,B,D,E), we observed that the overexpression of FGFs/CCND1 markedly increased the cancer cell proliferation (Figures 1C,F). To determine the sensitivity to gefitinib, we measured the IC50 for gefitinib of these cells. Strikingly, overexpression of FGF3/4/19/CCND1 increased the IC50 by 18 times and 32 times in PC-9-4X and HCC827-4X, respectively (Figures 1G,J). Colony staining was performed to confirm the resistance induced by FGFs/CCND1 (Figures 1H,I,K,L). Taken together, our data indicated that amplification of FGFs/CCND1 indeed caused gefitinib resistance.

### Screening of FGFR Inhibitors for Overcoming Gefitinib Resistance Induced by FGFs/CCND1

**FIGURE 2:**
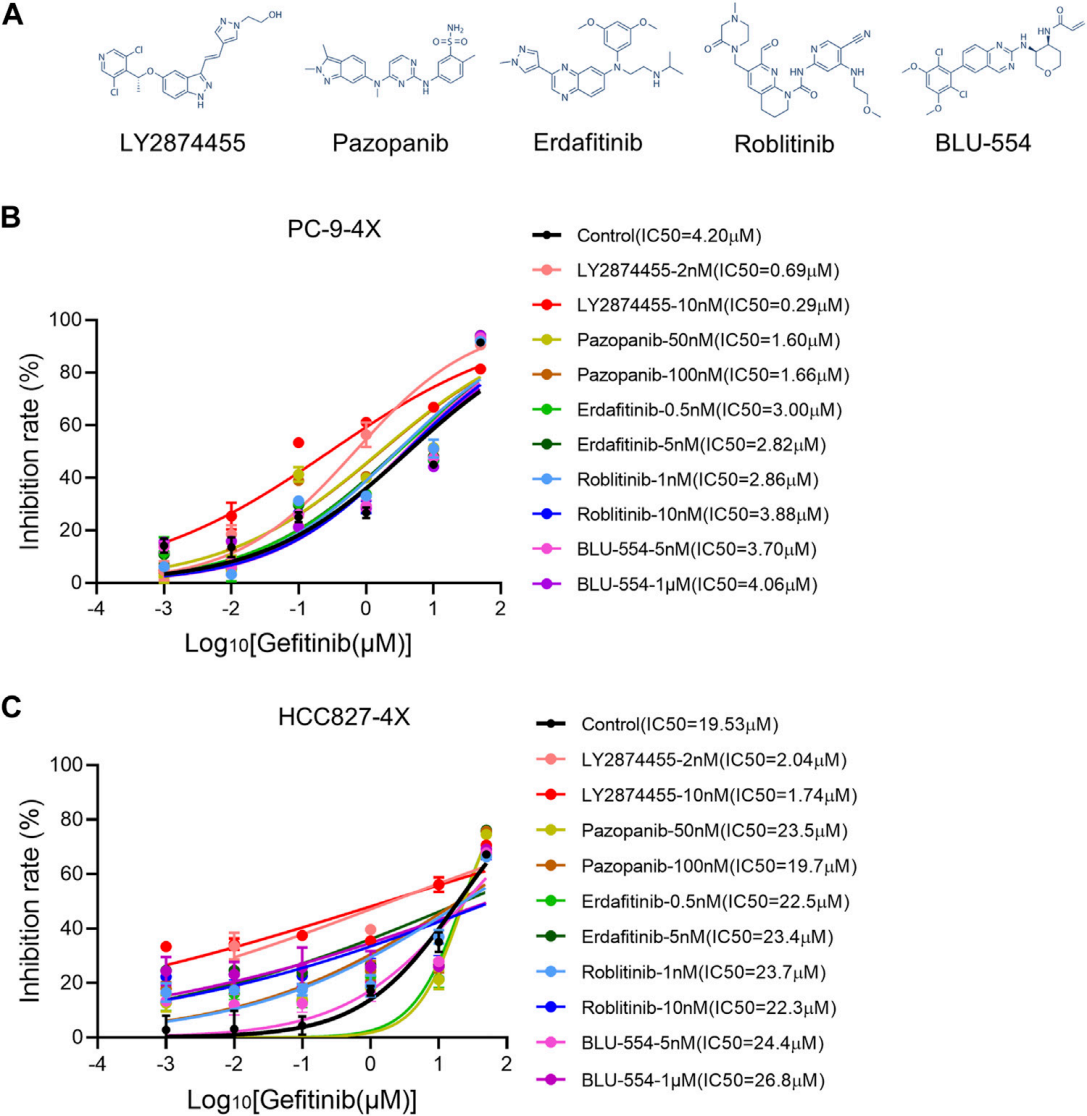
Screening of FGFR inhibitors for overcoming gefitinib resistance induced by FGFs/CCND1. **(A)** The structural formulae of FGFR inhibitors. **(B,C)** PC-9-4X and HCC827-4X cells were treated with gefitinib combined with each FGFR inhibitor for 72 h. The inhibition rate was detected by the CCK8 assay. Error bars indicate SD.

Next, we tested different FGFR inhibitors (including LY2874455, pazopanib, erdafitinib, roblitinib, and BLU-554-5) to find out the optimal FGFR inhibitor(s) for overcoming gefitinib-resistant cells (Figure 2A). As shown in Figure 2B, roblitinib and BLU-554 decreased the IC50 for gefitinib in PC9-4X cells, while the effect was not concentration-dependent. On the other hand, roblitinib and BLU-554 both slightly increased the IC50 for gefitinib in HCC-827-4X cells (Figure 2C). Notably, LY2874455 dramatically decreased the IC50 for gefitinib in both PC9-4X and HCC827-4X cells at 2 and 10 nM, and the effects were concentration-dependent (Figures 2B,C). Pazopanib at 50 and 100 mM significantly decreased the IC50 for gefitinib in PC9-4X cells, but not in HCC827-4X cells (Figures 2B,C). Erdafitinib concentration-dependently reduced IC50 for gefitinib in PC9-4X cells but increased the IC50 for gefitinib in HCC827-4X cells (Figures 2B,C). Taken together, we found that LY2874455 was the most effective inhibitor to reverse the FGFs/CCND1 induced gefitinib resistance in different cell lines; thus, it was selected for subsequent studies.

### The Combination of Gefitinib/LY2874455/Abemaciclib Reverses the Gefitinib Resistance Induced by FGFs/CCND1 *In Intro*

**FIGURE 3:**
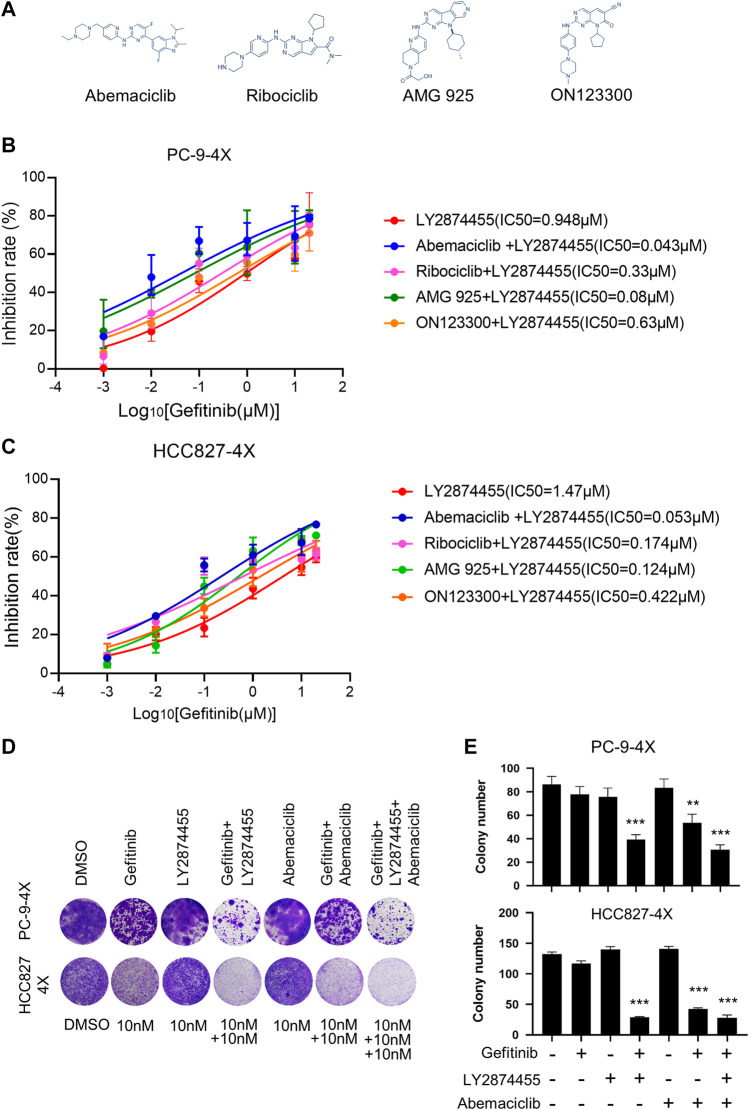
The combination of gefitinib/LY2874455/abemaciclib reverses the gefitinib resistance induced by FGFs/CCND1 *in vitro.* **(A)** The structural formulae of inhibitors target to CDK4/CCND1 complex. **(B)** PC-9-4X Cells were treated with LY2874455 and gefitinib in combination with different CDK4 inhibitors for 72 h to measure the inhibition rate by CCK8. Error bars represented SD. **(C)** HCC827-4X cells were treated with LY2874455 and gefitinib in combination with different CDK4 inhibitors for 72 h to measure inhibition rate by CCK8. Error bars represented SD. **(D)** Crystal violet staining of PC-9-4X or HCC827-4X cell lines after treatment with DMSO or the indicated drugs for 14 days. **(E)** The number of stained cells in crystal violet staining in **(D)**. Error bars represented SD (*p*-values reflected comparison to the control samples. *p < 0.05. **p < 0.01. ***p < 0.001, one-way ANOVA, with Tukey’s test).

We next tested if the addition of inhibitors targeting the CCND1/CKD4 complex could further reduce the IC50 for gefitinib in PC9-4X and HCC827-4X cells (Figure 3A). Cells were treated with LY2874455 and gefitinib in combination with different CCND1/CKD4 inhibitors, and the results showed that the IC50 for gefitinib in the abemaciclib + LY2874455 group was much lower than that in other groups (Figures 3B,C), indicating that abemaciclib exhibited the best suppression activity and was selected for further examination. The colony formation assay showed that single inhibitor treatment of gefitinib, LY2874455, or abemaciclib failed to inhibit the cell growth of PC9-4X or HCC-827-4X cells (Figures 3D,E). However, abemaciclib in combination with gefitinib significantly attenuated cell growth of PC9-4X and HCC-827-4X cells (Figures 3D,E), and the inhibitory effect was further enhanced by LY2874455 (Figures 3D,E).

### The Activity of the Epidermal Growth Factor Receptor, Mammalian Target of Rapamycin, and Mitogen-Activated Protein Kinase in Response to Combination Treatment of Gefitinib/LY287445/Abemaciclib in PC-9-NC and PC-9-4X Cells

**FIGURE 4:**
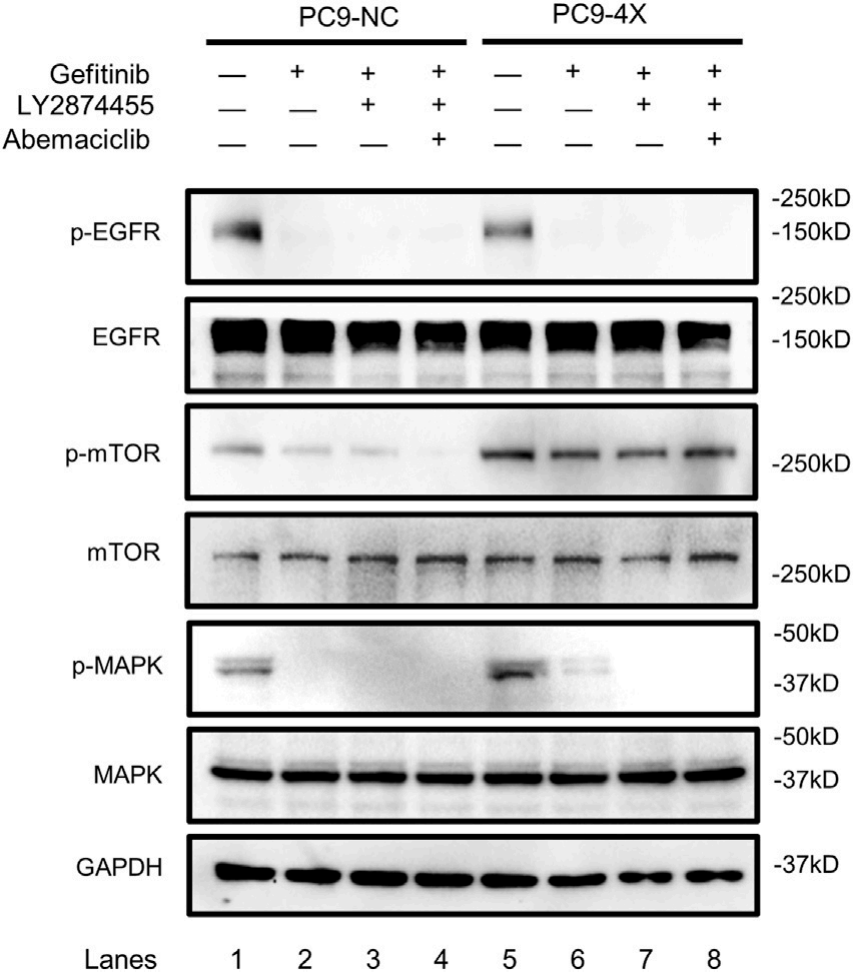
The activity of the EGFR, mTOR, and MAPK in response to gefitinib (10 nM) and/or combined treatment with LY287445 (10 nM) and/or abemaciclib (10 nM) in PC-9-NC and PC-9-4X cells. The cells were treated for 24 h before the Western blot analysis. GAPDH was used as a loading control.

FGF/FGFR exhibits its physiological functions by triggering various downstream signaling pathways, including RAS/MAPK and PI3K/AKT/mTOR (Xie et al., 2020). In order to find out which signaling pathway is activated in the FGF3/4/19/CCND1 amplification model, we tested the phosphorylation levels of the main components of these pathways. As shown in Figure 4, the phosphorylation of EGFR was decreased when the PC-9-4X cells were treated with gefitinib alone (lanes 2 and 6) or combined with other inhibitors (lanes 3, 4, 7, and 8), indicating that the activity of EGFR was not essential for gefitinib-resistant cells. Interestingly, the phosphorylation of MAPK was increased in PC-9-4X cells compared with PC-9-NC cells (Figure 4, lanes 1 and 5). Importantly, with the treatment of gefitinib, the MAPK activity in PC-NC was diminished. However, PC-9-4X still exhibited MAPK activity (Figure 4, lanes 2 and 6), suggesting that FGFs/CCND1 induced enhanced cell proliferation and gefitinib-resistant *via* the MAPK pathway. The activation of MAPK was abolished with the treatment of LY2874455 or/and abemaciclib (Figure 4, lanes 3, 4, 7, and 8). Interestingly, the activity of mTOR was also increased in PC-9-4X compared with PC-9-NC, yet the activity could not be inhibited by LY2874455 or/and abemaciclib treatments, indicating that the reversal of gefitinib resistance was independent of the mTOR pathway in PC-9-4X.

### The Combination of Gefitinib/LY2874455/Abemaciclib Reverses the Gefitinib Resistance Induced by FGFs/CCND1 *In Vivo*

**FIGURE 5:**
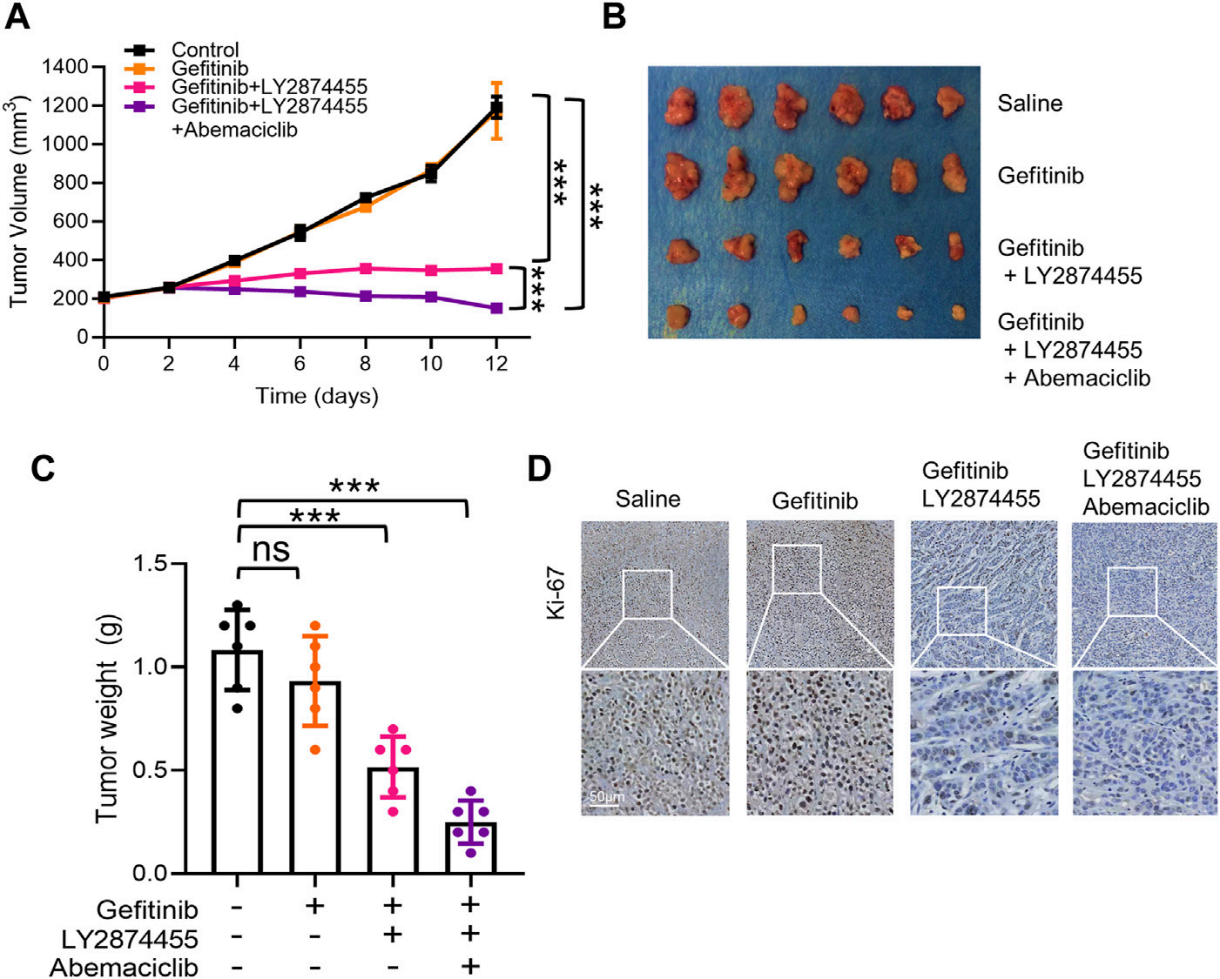
The combination of gefitinib/LY2874455/abemaciclib reverses the gefitinib resistance induced by FGFs/CCND1 *in vivo.* **(A)** Mice bearing PC-9-4X were injected twice a week with gefitinib (100 mg/kg) combined with LY2874455 (3 mg/kg) or abemaciclib (50 mg/kg) or PBS by oral administration, and tumor growth was monitored for 2 weeks during treatments (*** *p* < 0.001, two-way ANOVA, with Tukey’s test). Profiles of tumor growth during the drug treatment are shown. For each treatment group, data are presented as mean tumor volume (mm^3^) ± SD, *n* = 6. **(B)** Tumors resected from each group with indicated treatment at the end of experiments are shown *n* = 6. **(C)** Tumor volume was measured using calipers. Mean ± SD tumor volumes on day 12 are shown. Each group contained 6 mice (ns, *p* > 0.05. ***p < 0.001, one-way ANOVA, with Tukey’s test). **(D)** Immunohistochemical staining with Ki-67 antibodies in xenograft tumors from each group. Scale bar, 50 mm.

To determine the effects of combined treatment of gefitinib/LY2874455/abemaciclib *in vivo*, a cell line-derived xenograft (CDX) model of NSCLC was generated by implanting PC-9-4X cells into nude mice. As shown in Figure 5, gefitinib alone failed to attenuate the tumor growth of PC-9-4X tumor in comparison with the control group. These results were consistent with our data *in vitro* that FGFs/CCND1 indeed could induce gefitinib resistance (Figure 5A). Co-treatment with LY2874455 and gefitinib significantly repressed the tumor growth and decreased the tumor weight compared to the gefitinib and control group (Figures 5A–C). In addition, tumor growth was further repressed in the gefitinib + LY2874455 + abemaciclib group (Figures 5A–C). The reduced expression of Ki67 from IHC staining was also observed in xenograft tumors when treated with LY2874455 or/and abemaciclib combined with gefitinib (Figure 5D).

## Discussion

The deregulation of bypass pathway signaling plays an essential role in TKI-resistant NSCLC (Lim et al., 2018; Westover et al., 2018; Wu and Shih, 2018). Here, we demonstrated that amplification of FGF3/4/19/CCND1 is associated with acquired TKI resistance in NSCLC. Around 3% of TKI resistance patients carried FGF3/4/19 or CCND1 amplification. FGF3/4/19/CCND1 overexpression markedly promoted cell proliferation and gefitinib resistance. Phosphorylation of MAPK was enhanced by FGFs/CCND1 overexpression. Significantly, treatments with gefitinib combined with FGFR inhibitor LY2874455 and CCND1/CDK4 inhibitor abemaciclib induced strong and synergistic growth suppression in FGF3/4/19/CCND1-overexpressing gefitinib-resistant NSCLC cells *in vitro* and xenografts *in vivo*. Finally, the activity of the MAPK signaling pathway was abolished by the drug combinations. Our study is the first to report a correlation between copy-number gain of FGF3/4/19/CCND1 and resistance to TKI inhibitors in NSCLC. Moreover, we provide a promising therapeutic strategy to reverse gefitinib resistance.

In order to survive, cancer cells activate the bypass signaling against apoptosis and cell cycle arrest when being treated by targeted therapy. Studies found that EGFR inhibitors upregulated several FGFs/FGFRs (including FGF2, FGF9, FGF13, FGFR1, FGFR2, and FGFR3) (Yue et al., 2021). Thus, it is possible that the frequency of FGF 3/4/19 amplification would be increased in EGFR-TKI-treated patients. Further studies would test this possibility. The FGF/FGFR network is a complex system that provides an autocrine receptor tyrosine kinase-driven bypass pathway that can induce drug resistance *via* multiple conventional downstream cascades (including PI3K, MAPK, AKT, and mTOR) (Xie et al., 2020). For instance, FGF3/4/19 promotes resistance to HER2 inhibitors lapatinib and trastuzumab in breast cancer (Hanker et al., 2017). Since increased FGF3/4/19 copy number is frequently detected and highly related to the initiation and progression of many tumors including urothelial carcinoma, multiple myeloma, prostate cancer, and hepatocellular carcinoma (Xie et al., 2020), we believe that our finding will also benefit other anti-cancer therapies targeting FGF3/4/19 variated tumors.

LY2874455 is a highly selective pan-FGFR inhibitor (against FGFR1-4) (Dehghanian and Alavi, 2021; Wu et al., 2018). Since all FGF ligands exert their functions through these four FGFRs, it was not surprising that LY2874455 could effectively reverse the gefitinib resistance in our study. Erdafitinib is another FGFR inhibitor that exhibits similar IC50 for FGFR(1–4) as LY2874455 (Perez-Garcia et al., 2018; Loriot et al., 2019); however, it only reversed the gefitinib resistance in PC9-4X, but not in HCC827-4X cells. Thus, the underlying mechanism warrants further investigation. Roblitinib, a reversible-covalent inhibitor of the kinase activity of FGFR4 (Fairhurst et al., 2020), failed to reverse the acquired gefitinib resistance in PC9-4X and HCC827-4X cells. Consistently, BLU-554, a highly selective irreversible inhibitor of FGFR4 (Kim et al., 2017), showed similar actions as rolitinib. These results suggest that the inhibition of FGFR4 may not be sufficient to reverse the gefitinib resistance mediated by FGF3/4/19/CCND1. Owing to the complex network of the FGF/FGFR system, we believe that it is more appropriate to treat the tumor with pan-FGFR inhibitors to completely block FGF/FGFR signaling.

Abemaciclib is a highly selective inhibitor of CDK4/CCND1 (IC50 = 2 nmol/L) and CDK6/CCND1 (IC50 = 10 nmol/L) complexes (Patnaik et al., 2016). In the present study, abemaciclib was the most effective agent to reverse gefitinib resistance in PC9-4X and HCC287-4X cells when combined with LY2874455 and gefitinib. CCND1 is a well-recognized human oncogene, and it plays a critical role in therapeutic resistance in many cancers (Chen et al., 2020). In addition, CCND1 amplification was recently found to be associated with a poor prognosis to immune checkpoint inhibitors (Schaer et al., 2018). Thus, although we observed that LY2874455 has an extraordinary effect on inhibiting FGFs/CCND1 amplification, it is essential to treat the cancer cells with CCND1 inhibitors to prevent acquired resistance through activation of CCDN1.

Of note, the MAPK signaling was activated in the PC9-4X cells, and gefitinib treatment could only completely abolish the activity of MAPK signaling in PC9-NC cells, but not in the PC-9-4X cells, while LY2874455 or in combination with abemaciclib completely abolished the activity of MAPK signaling. The aforementioned results indicated that LY2874455 alone or in combination with abemaciclib reversed gefitinib resistance *via* repressing the MAPK signaling pathways. It should be considered that mTOR signaling in PC-9-4X was not completely blocked by our therapeutic strategy, and future study will clarify this issue for optimized treatment.

In this study, several limitations should be carefully considered. First, since the FGF3/4/19 genes are closely located on chromosome 11q13 and are amplified simultaneously in NSCLC, we did not investigate the contribution of each gene to gefitinib resistance. Second, our study was performed *in vitro* and *in vivo* assays based on cellular and animal models, and further studies should conduct clinical trials.

In conclusion, the present study for the first time established the FGF3/4/19/CCND1-amplification NSCLC cell lines, which exhibit gefitinib resistance. Further screening studies revealed that LY2874455 and abemaciclib showed superior inhibition of NSCLC cell growth both *in vitro* and *in vivo* in the gefitinib-resistance models. Mechanistically, we found that FGFs/CCND1 could activate MAPK, but not the mTOR pathway, which was abolished by the combination drugs. Collectively, our data provide a strong rationale for clinical testing of dual targeting of FGFR and CCND1/CDK4 in NSCLC patients with FGF3/4/19/CCND1-amplification tumor resistant to gefitinib. Finally, since FGF3/4/19/CCND1 genes are frequently amplified in many solid cancers, it is worthy to test this therapeutic strategy in other cancers beyond NSCLC.

## Data Availability

The original contributions presented in the study are included in the article/Supplementary Materials; further inquiries can be directed to the corresponding authors.

## Supplementary Material

Supplementary Figure S1 Summary of mechanisms of resistance to different generation EGFR TKIs. **(A)** The pie chart depicts resistance mechanisms that have been identified after resistance to first- and second-generation EGFR TKIs. **(B)** The resistance mechanisms of resistance to third-generation EGFR TKIs. **(C)** The mechanisms of resistance to all EGFR TKIs after combining the data from **(A, B)**.
