# Supplementary figures and images for: LY2874455 and Abemaciclib Reverse FGF3/4/19/CCND1 Amplification Mediated Gefitinib Resistance in NSCLC
Source: Front Pharmacol. 2022 Jun 23;13:918317. doi: 10.3389/fphar.2022.918317 (PMC9260114; doi:10.3389/fphar.2022.918317)

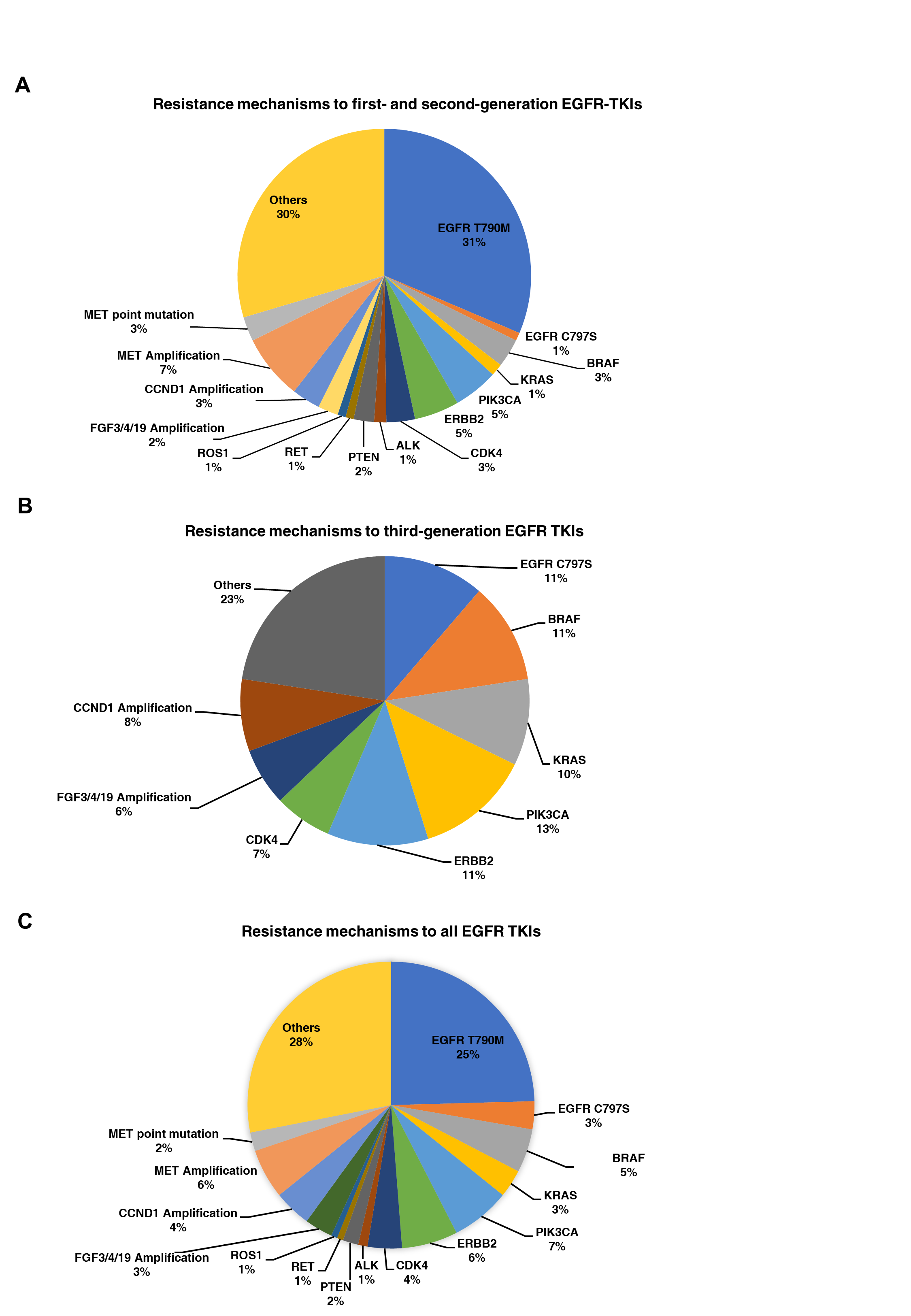

Supplement: Supplementary file 1 [file Image1.tif]
